# The Current and Emerging Role of Statins in the Treatment of PCOS: The Evidence to Date

**DOI:** 10.3390/medicina60020244

**Published:** 2024-01-30

**Authors:** Tea Shehu Kolnikaj, Rok Herman, Andrej Janež, Mojca Jensterle

**Affiliations:** 1 Department of Endocrinology, Diabetes and Metabolic Diseases, University of Medicine Tirana, 1000 Tirana, Albania; tkolnikaj@yahoo.com; 2 Department of Endocrinology, Diabetes and Metabolic Diseases, University Medical Center Ljubljana, 1000 Ljubljana, Slovenia; rokherman2@gmail.com (R.H.); andrej.janez@kclj.si (A.J.); 3 Department of Internal Medicine, Faculty of Medicine, University of Ljubljana, 1000 Ljubljana, Slovenia

**Keywords:** polycystic ovary syndrome, steroidogenesis, hyperandrogenemia, statins, dyslipidemia

## Abstract

Polycystic ovary syndrome (PCOS) manifests a multifactorial pathology characterized by polycystic ovaries, menstrual cycle disorders, varying degrees of hyperandrogenism, and an ad-verse metabolic risk profile. The position of hyperandrogenism in this syndrome has been extensively studied. A multitude of mechanisms place it in the position of cause but also of consequence; therefore, ongoing research efforts are focused on identifying medications that can effectively reduce levels of androgens in women with PCOS. Moreover, lipid abnormalities are common in this population, with up to 70% of patients having dyslipidemia. Statins may have potential therapeutic benefits for women with PCOS, as they have been shown to improve insulin resistance and reduce the risk of cardiovascular disease. In addition, their role in accelerated steroidogenesis by limiting one source of cholesterol, influencing enzymatic activity, and providing several other beneficial mechanisms is widely investigated. This review aimed to provide a comprehensive overview of the pathogenesis of androgen excess and dyslipidemia in PCOS, as well as the therapeutic potential of statins.

## 1. Introduction

Polycystic ovary syndrome (PCOS) is a multifactorial endocrine disorder characterized by various underlying abnormalities, such as enlarged ovaries with numerous follicles, ovulatory dysfunction, insulin resistance, and hyperandrogenism. Modified Rotterdam criteria for PCOS diagnosis include the presence of at least two out of three of the following manifestations: signs of clinical or biochemical hyperandrogenemia, chronic ovulatory dysfunction, and polycystic ovaries on ultrasound, after the exclusion of secondary causes [1]. Depending on the presence or absence of these cardinal features, a case of PCOS is then classified as phenotype A, B, C, or D. Phenotype A includes hyperandrogenism, ovulatory dysfunction, and polycystic ovaries; Phenotype B includes hyperandrogenism and ovulatory dysfunction; Phenotype C includes hyperandrogenism and polycystic ovaries; Phenotype D includes ovulatory dysfunction and polycystic ovaries [2]. Its etiology remains unclear; however, solid evidence supports the interplay between genetic and environmental factors [3]. A critical aspect of our understanding of this syndrome is elucidating the interplay of underlying mechanisms that contribute to hyperandrogenemia, as well as its pivotal role in the pathogenesis and progression of the disorder.

Increased levels of androgens originate mainly from the ovaries. They are related to a hyperplasic theca compartment and the excessive expression of several genes involved in steroidogenesis [4,5]. In addition, adrenal glands are another important place of androgen production [6,7,8,9]. The overproduction from ovaries and adrenal glands works together with the increase in the bioavailable fraction due to a decrease in sex hormone binding globulin (SHBG). This combination gives a specific clinical picture of hyperandrogenism [10]. The most typical clinical manifestations include hirsutism, acne, and androgenic alopecia. Other manifestations, such as weight gain, menstrual irregularities, acanthosis nigricans, and insulin resistance, are also in direct correlation with increased androgen levels [11]. The importance of ovarian dysfunction and consequential hyperandrogenemia in PCOS, combined with the fact that PCOS can even be reversed in some women by reducing circulating androgen levels, emphasizes the need to understand this abnormality and potentially use it as a treatment target in some patients [11].

Androgens stimulate and promote PCOS by inducing cellular activities such as apoptosis, autophagy, mitochondrial dysfunction, and endoplasmic reticulum stress in granulosa cells and oocytes [12]. The role of hyperandrogenemia in the development of PCOS is further implied by the increased incidence of PCOS in women who have faced prenatal androgen exposure [5]. Furthermore, increased androgens add to other systemic effects such as cardiovascular disease (CVD), type 2 diabetes mellitus, kidney disease, and obesity [12].

To a large degree, the management of PCOS patients depends on symptomatic treatment. Management goals should be set collaboratively with patients. All should be counseled on developing or maintaining a healthy lifestyle, including a diet with all the components of healthy eating behaviors, activity, and stress management [13]. Hormonal therapies primarily address endometrial health and reduce mild to moderate symptoms of hyperandrogenism. Metformin is prescribed to moderately decrease testosterone and hyperandrogenic symptoms and prevent diabetes in adults. Additionally, it can also contribute to weight loss [14]. In PCOS patients with obesity, early data suggest that GLP-1 receptor agonists are a promising treatment option, and bariatric surgery is highly effective for a limited number of patients [6,7,8,9]. Used medications that predominantly target androgen excess are oral contraceptives, spironolactone, cyproterone acetate, flutamide, and finasteride [15].

Some studies have suggested that statins may have potential therapeutic benefits for women with PCOS, as they have been shown to improve insulin resistance and reduce the risk of CVD in this population [15]. In addition, their role in accelerated steroidogenesis by limiting one source of cholesterol and providing several other beneficial mechanisms is widely investigated [16]. 

This narrative review aims to provide a comprehensive overview of the pathogenesis of androgen excess and its pathophysiological role in PCOS. Furthermore, the role of dyslipidemia in clinical presentation is explored alongside the potential of lipid-lowering medications to alleviate hyperandrogenism and provide other beneficial effects. The last chapter is dedicated to future perspectives on the topic.

## 2. Pathophysiology of Androgen Excess

Androgen excess constitutes one of the cardinal features of PCOS [17]. In addition to being a key diagnostic criterion, hyperandrogenism also serves as a primary driver of diverse clinical manifestations. Elevated androgen levels are implicated in the disruption of normal ovarian function, leading to irregular menstruation and infertility. Clinically, hyperandrogenism is often manifested as hirsutism, severe acne, and androgenic alopecia, which not only contribute to the physical burden of PCOS but also to significant psychosocial distress. Moreover, effects of androgen excess extend beyond reproductive anomalies, exerting metabolic changes that increase the risk of insulin resistance, type 2 diabetes, and cardiovascular complications [5].

Testosterone and androstenedione levels are reported to be elevated in about 60–80% of women with PCOS [16] and are in correlation with increased levels of luteinizing hormone (LH) [18]. Pathophysiologically, androgen excess can disrupt the normal negative feedback effect of estradiol and progesterone on gonadotropins, predominantly LH, leading to LH increase [19,20]. This disruption can also occur indirectly by modulating GABAA receptors [21]. Moreover, hyperandrogenemia worsens central adiposity; therefore, more testosterone is converted into estrone, consequently increasing the LH/follicle stimulating hormone (FSH) ratio [22]. Moreover, hyperandrogenemia possesses additional effects. It is strongly related to insulin resistance through direct mechanisms on insulin sensitivity and the expression of GLUT4, as well as insulin clearance. It also reduces insulin-like growth factor 2 (IGF-II) and upregulates anti-Müllerian hormone (AMH), both of which are positively related to follicle diameters [22]. Furthermore, hyperandrogenemia is responsible, at least partially, for increased inflammation and oxidative stress [23]. Lastly, hyperandrogenism also presents an additional risk factor for cardiovascular disease in women with PCOS. A recent meta-analysis suggested a link between androgen levels in PCOS and an increase in total cholesterol coupled with a decrease in HDL-C levels, while triglyceride and LDL-C levels remain unaffected [24]. Moreover, PCOS patients with marked hyperandrogenism may experience microvascular dysfunction, characterized by compromised vasodilation capacity. Studies have indicated a direct relationship between endothelin-1 levels and free testosterone [25]. However, the evidence remains inconclusive regarding endothelial dysfunction, as measured by dynamic tests, and its association with hyperandrogenism in PCOS [26,27].

The majority of evidence suggests that the ovaries are the primary source of increased androgens in PCOS [28]. However, adrenal contributions cannot be overlooked, as a significant subset of women with PCOS also display abnormal levels of adrenal androgens [29]. Distinguishing between these sources is clinically relevant, as it may influence treatment choices and prognosis. Additionally, recent studies utilizing advanced imaging techniques have shown that adrenal morphology and function are altered in PCOS, suggesting a more significant role of adrenal-derived androgens than previously understood [30]. Beyond ovarian and adrenal output, key additional contributions come from the conversion of DHEA to androstenedione, androstenedione to testosterone, and testosterone to dihydrotestosterone within adipose tissue, augmenting the pool of circulating androgens. Although clinical manifestations are usually similar regardless of the exact source of excess androgen, elevated DHEAS levels, particularly in hyperandrogenic states, are associated with a lower body weight, decreased insulin levels, and an improved metabolic profile [11,31].

The hyperandrogenic phenotype is primarily familiar, suggesting that genetic factors, especially maternal inheritance, could govern steroidogenesis. Moreover, even prenatal exposure to maternal androgens precipitates PCOS, showcasing hyperandrogenemia as an environmental and genetic factor in defining the etiology of this syndrome. The relative contribution of androgen production between the adrenal gland and ovaries is a topic of frequent inquiry. Data on selective ovarian androgen suppression by long-acting gonadotropin-releasing hormone (GnRH) agonists suggests that ovaries are the principal source of excessive androstenedione and testosterone in PCOS [11,32]. However, a small number of patients present with functional adrenal hyperandrogenemia, implicating the dysregulation of steroidogenesis in the zona reticularis, similar to that of theca cells in ovaries [33]. Among patients with hyperandrogenism, there are variable expressions of this characteristic feature [34].

**Figure 1:**
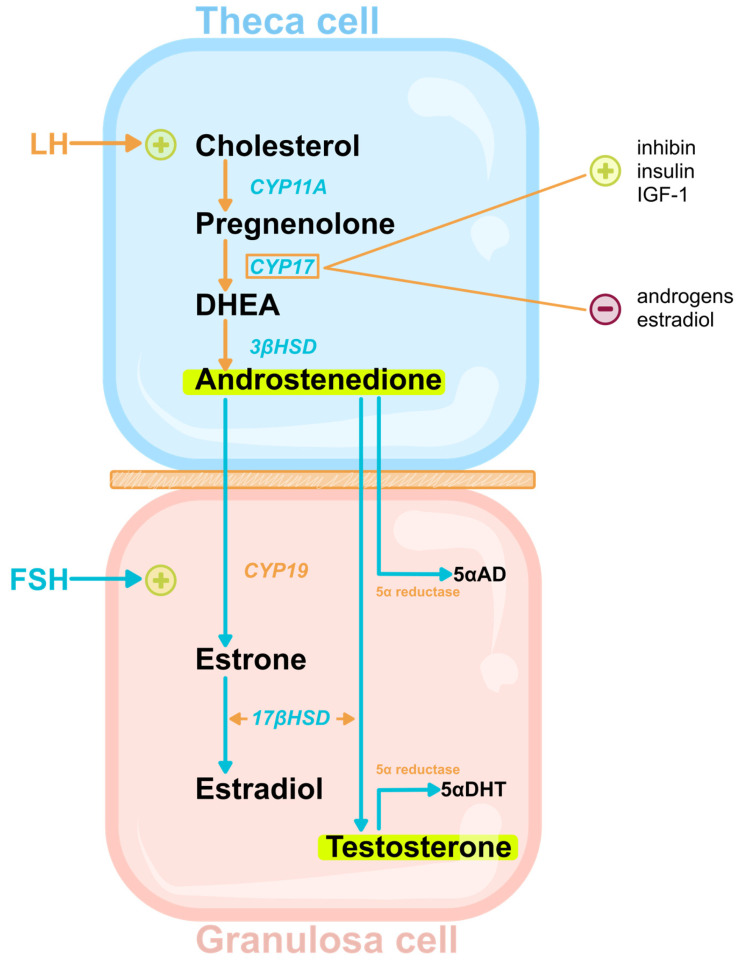
Steroidogenesis in PCOS. Legend: CYP11A—cholesterol side-chain cleavage enzyme; CYP17—cytochrome P450 17α-hydroxylase/17,20-lyase; 3βHSD—3β-hydroxysteroid dehydrogenase; IGF1—Insulin-like Growth Factor 1; 5αAD—5α-androstane-3,17-dione; CYP19—aromatase; 17βHSD—17β-hydroxysteroid dehydrogenase; LH—luteinizing hormone; FSH—follicle-stimulating hormone; DHEA—dehydroepiandrosterone; 5αDHT—5α-dihydrotestosterone.

Androgen position in PCOS pathogenesis turns us back to ovarian steroidogenesis and its specifics in PCOS in order to understand the differences with normal steroidogenesis. Ovarian follicle steroidogenesis is accomplished by the cooperation of a two cell/two hormone system—theca and granulose cells/LH and FSH—with specific characteristics in PCOS, as demonstrated in Figure 1. The first and rate-limiting step in steroidogenesis is the conversion of cholesterol to pregnenolone by the cholesterol side-chain cleavage enzyme, P450scc (CYP11A1), a cytochrome P450 (CYP) enzyme that lies on the inner mitochondrial membrane. Under the influence of LH, theca cells produce androstenedione de novo through the main androgen-synthesis enzyme CYP17. This enzyme converts pregnenolone to dehydroepiandrosterone (DHEA). Another enzyme is 3β-hydroxysteroid-dehydrogenase (3β-HSD), which converts DHEA to androstenedione. Very low levels of testosterone are produced in theca cells because of the low level of expression of 17β-hydroxysteroid-dehydrogenase (17β-HSD), which converts androstenedione to testosterone. In granulosa cells, androstenedione is converted mainly into estradiol through aromatase (CYP19). However, in PCOS, CYP17 is overexpressed, and androstenedione is the major androgen produced. CYP17 activity is reinforced by insulin, insulin-like growth factor-1 (IGF-1), and inhibin, which are all elevated in PCOS patients. Its inhibition by androgens and estradiol is also impaired by the decreased conversion of androstenedione into estradiol because of the diminished activity of aromatase. Androstenedione is then metabolized to testosterone through 17β-HSD or to 5α-androstane-3,17-dione (5α-AD) through 5α-reductase, which is another overexpressed enzyme in PCOS. Testosterone is converted to 5α-dihydrotestosterone (5α-DHT) again through 5α-reductase [35,36,37,38,39].

Therefore, in PCOS, theca cells have an increased steroidogenic capacity and increased responsiveness to hCG with consequential higher androgen production. They are considered the most important place of androgen synthesis in PCOS, even though testosterone is produced in the granulosa cells [40,41]. In addition, an imbalance in the hypothalamic–pituitary–ovarian axis with an increase in LH/FSH ratio causes the arrest of theca cells in preantral and antral stages and facilitates theca cell hyperplasia with more layers of differentiated steroidogenic cells [42]. This hyperplasia is associated with follicular fluid accumulation forming a pearl-like appearance. Furthermore, hyperinsulinemia mimics the trophic action of LH on theca cells, acting as a co-gonadotropin with LH, and it also mediates its pulses [43,44]. LH receptors in normal ovaries possess the ability of desensitization during folliculogenesis at the time of LH rise, but animal studies have demonstrated even the up-regulation of LH receptors, further potentiating androgen synthesis [45]. Moreover, decreased aromatase (CYP19) activity further upregulates androgen production [5].

On the other side, granulosa cells are underdeveloped, with fewer-than-expected potential estrogen-producing cells. Larger dominant follicles normally express high concentrations of aromatase and nearly undetectable 5α-reductase activity. In PCOS, where follicles remain arrested in the preantral/antral phase, aromatase activity is low, whereas 5α-reductase activity remains high. As a result, there is an accumulation of androstenedione, and the concentration of 5α-AD is elevated [46,47,48]. Estrogen levels are not reduced because of peripheral tissue conversion from androstenedione to estrone, and combined with the increased LH/FSH ratio, they can even promote malignant tumors of the reproductive system [49]. In addition, granulosa cells luteinize prematurely due to androgen and insulin excess [46]. This premature luteinization causes granulosa cells to respond to LH and to produce an excessive amount of progesterone compared to cells of normal ovaries in similar stages. Progesterone produced by theca cells is also elevated because of increased steroidogenic capacity [50]. On the other hand, AMH levels are elevated, indicating the number of growing follicles and reflecting intrafollicular androgenic status because androgens stimulate the early phases of follicular growth [51].

Other disruptors also enhance this abnormal steroidogenesis in PCOS. Advanced glycation end products (AGEs), highly reactive molecules formed after the glycation of lipids and proteins, are elevated. They are associated with hyperandrogenemia through the alteration of the activity of different enzymes, such as cholesterol side-chain cleavage enzyme cytochrome P450, steroidogenic acute regulatory protein, 17α-hydroxylase, and 3β-hydroxysteroid dehydrogenase. They also affect the expression of the LH and AMH receptors and their signaling pathways in granulosa cells [52]. Moreover, systemic and tissue-specific inflammation are common characteristics of obesity and PCOS [53]. Pro-inflammatory cytokines and reactive oxygen species alter cyclicity, steroidogenesis, and ovulation. Gut microbiome dysbiosis also contributes to inflammation, steroidogenesis, and the expression of mRNAs in the oocyte [54].

To conclude, polycystic ovaries produce increased concentrations of androgens and progesterone and abnormally high concentrations of 5α-reduced androgens compared to normal ovaries due to the disruption of normal steroidogenesis at multiple points.

## 3. Cardiovascular Risk and Dyslipidemia in PCOS

Premenopausal women with PCOS display a distinct cardiovascular and metabolic risk profile, as well as evidence of accelerated subclinical atherosclerosis. However, this risk does not consistently translate to an increased rate of cardiovascular events in the postmenopausal period. Studies have documented various preclinical changes in arterial structure among PCOS women of reproductive age [55]. A thorough systematic review and meta-analysis, encompassing 96 studies with 5550 PCOS patients and 5974 controls, sought to assess PCOS’s impact on early indicators of atherosclerosis, using measurements like carotid intima-media thickness (cIMT), flow-mediated dilation (FMD), nitroglycerin-induced dilation (NMD), pulse wave velocity (PWV), and coronary artery calcium (CAC) [55]. PCOS patients exhibited notably thicker cIMT, impaired FMD and NMD, increased PWV, and a higher rate of CAC compared to controls [55,56]. Yet, the link between PCOS, clinical atherosclerosis, and actual cardiovascular events is more variable. Certain research indicates no heightened risk for major vascular diseases, abdominal aortic plaque, myocardial infarction (MI), or stroke [57,58,59,60]. A 2017 meta-analysis of eight studies including 128,977 women aged 36–71, over a follow-up of 10–40 years, indicated a significant correlation between PCOS and stroke risk [61], which diminished and became statistically insignificant after BMI adjustments [61]. Another meta-analysis of five case–control and five cohort studies involving over 100,000 women aged 20–74 years, followed for 7–40 years, found no substantial link between PCOS and MI [62]. On the other hand, additional systematic reviews and meta-analyses evaluated the incidence of cardiovascular and cerebrovascular events in PCOS patients, reviewing 10 cohort studies with 166,682 participants followed for over 10 years on average. The findings revealed an elevated risk of cardiovascular diseases and cerebrovascular incidents, including MI, ischemic heart disease, and stroke in women with PCOS [63]. In addition, other studies have reported an increased incidence of non-fatal cerebrovascular disease [64] and a higher risk of coronary heart disease in PCOS women, even after BMI adjustments [65,66].

Metabolic abnormalities are common in PCOS patients, including obesity, insulin resistance, and dyslipidemia [67].These abnormalities can act as vital risk factors, increasing the chances of developing type 2 diabetes, coronary heart disease, and stroke [68]. According to the National Cholesterol Education Program (NCEP) guidelines, approximately 70% of PCOS patients exhibit abnormal serum lipid levels [69]. Dyslipidemia is characterized by elevated levels of low-density lipoprotein (LDL) and triglycerides, low levels of high-density lipoprotein (HDL), and increased total cholesterol and lipoprotein concentrations, which collectively contribute to a high CVD risk profile [70,71]. In a meta-analysis by Wild et al., triglyceride concentration was 26 mg/dL higher, and HDL-cholesterol concentration was 6 mg/dL lower in women with PCOS compared to the controls. In addition, LDL-cholesterol and non-HDL-cholesterol concentrations were higher in the PCOS group, by 12 mg/dL and 19 mg/dL, respectively, and these results were irrespective of body mass index (BMI) [72]. A study by Conway et al. demonstrated that even lean PCOS patients carried an increased CVD risk with reduced HDL and HDL-2 concentration, which also indicates alterations in HDL quality [73]. Studies have also revealed that patients exhibit distinctive differences in the subtypes of LDL particles, including a higher proportion of small, dense LDL particles and a decreased mean LDL particle size. These quantitative and qualitative alterations may contribute to the increased CVD risk observed in this population [74].

The expression of different apolipoproteins on the surface of lipoproteins has also been a topic of research in PCOS. Table 1 presents different apolipoprotein levels and their characteristics in PCOS women.

Even though different studies reported no association between high testosterone levels and lipid profile [80], it has already been demonstrated that hepatic steatosis in PCOS patients is tightly related to hyperandrogenemia and an adverse metabolic profile [81]. Dyslipidemia could potentially contribute to hyperandrogenemia in several ways [80]. Hypomethylated genes involved in lipid and steroid synthesis may be involved in excess androgen production [82]. As mentioned in Table 1, apolipoprotein A1, which is low in PCOS women, is responsible for the accumulation of cholesteryl ester in steroidogenic cells and some steroidogenic enzymes. Reduced CYP19 expression due to apolipoprotein A1 depletion may be at least partially important for high testosterone levels because of an impeded conversion of testosterone to estradiol in granulosa cells [83]. On the other side, hyperandrogenemia acting interchangeably with insulin resistance can cause abnormal lipid metabolism accompanied by obesity, dyslipidemia, hypertension, nonalcoholic fatty liver disease, and sleep-disordered breathing [21]. Increased testosterone is associated with an increased clearance of HDL, with low levels commonly observed in patients. According to a recent meta-analysis, androgen excess in PCOS is associated with higher total cholesterol and lower HDL levels, with no impact on triglycerides and LDL levels [24]. The preliminary results of one study have shown a positive correlation between the abnormal dynamic tests of endothelial function and testosterone levels [26]. High androgen concentrations are also related to fat distribution (preferential intra-abdominal fat deposition and increased subcutaneous adipose storage) [84]. Schube et al. showed that high serum levels of triglycerides, free fatty acids, and oxidized LDL (oxLDL) resulted in mitochondrial dysfunction with the augmented release of ROS, leading to ovarian damage and an increased rate of follicular atresia. Lectin-like oxLDL receptor-1, toll-like receptor 4, and a cluster of differentiation 36, which are oxLDL receptors, and the oxLDL-dependent activation of these receptors can result in the apoptosis of human granulosa cells and disordered ovulation [85].

The heritability of dyslipidemia has been investigated in various populations of individuals with PCOS. Sam et al. reported that LDL levels are increased in affected sisters of women with PCOS, consistent with a heritable trait. In addition, the prevalence of metabolic syndrome is increased in affected sisters [86]. Sam et al. also found that mothers of women with PCOS had higher LDL-cholesterol levels, whereas triglycerides and HDL-cholesterol did not differ compared with control women. The predictors of LDL levels in mothers were their daughter’s LDL levels and their own unbound testosterone levels [87]. A study comparing a group of 80 sons of women with PCOS to 56 sons of the control group concluded that even sons of women with PCOS exhibit significantly higher LDL-cholesterol levels than those of the control group [88].

In conclusion, dyslipidemia is a frequently observed characteristic of PCOS and is associated with an atherogenic, heritable profile, as well as common comorbidities such as hyperandrogenemia and insulin resistance. Given the increased risk of CVD in PCOS patients, lipid profile testing is necessary in routine evaluation and management.

## 4. Statins for PCOS Management

Recent studies have investigated the potential of lipid-lowering medications, particularly statins, for reducing androgen levels in women with PCOS. While these studies did not have inclusion criteria specifically for dyslipidemia, the prevalence of lipid abnormalities in PCOS populations meant that most participants exhibited these characteristics. Although statins do not cause a decline in steroidogenic capacity or lower androgen levels compared to placebo in healthy individuals, studies in women with PCOS often demonstrate different effects [89]. Specific randomized control trials are summarized in Table 2. A meta-analysis that evaluated the impact of statins alone or combined with metformin in PCOS patients demonstrated a significant decline in total testosterone and free testosterone, DHES, androstenedione, LH, LH/FSH ratio, and prolactin in the statin group. This study also demonstrated a significant decline in total cholesterol, LDL cholesterol, triglycerides, fasting glucose, insulin sensitivity index, and high-sensitivity C-reactive protein in the statin group [90]. Another meta-analysis also demonstrated the beneficial effects of statins combined with metformin to lower testosterone levels, cholesterol, and triglycerides but no significant difference in HDL, FSH, prolactin, fasting glucose, and insulin sensitivity index compared to metformin alone [91]. A prospective, randomized crossover trial in 48 women with PCOS compared a combined oral contraceptive (COC) with simvastatin and COC alone in PCOS women. It concluded that simvastatin had additional effects in lowering androgens, improving the lipid profile and systemic inflammation markers [92].

Studies have also compared the effects of various statins on women with PCOS. A prospective, randomized trial of 64 women with PCOS compared atorvastatin and simvastatin. It was concluded that both statins were effective in reducing inflammation, hyperandrogenemia, oxidative stress, and improving metabolic parameters. While atorvastatin had more noticeable effects on fasting insulin and insulin sensitivity, simvastatin had a dominant effect on total testosterone [93]. Positive atorvastatin effects on insulin resistance have also been demonstrated in a meta-analysis [94]. However, atorvastatin’s effects on insulin resistance are not one-sided. In a prospective, randomized, double-blind, placebo-controlled study by Puurunen et al., 15 women with PCOS were treated with atorvastatin 20 mg/day versus 13 women with PCOS given a placebo for 6 months. They concluded that atorvastatin therapy improves chronic inflammation and lipid profile but impairs insulin sensitivity. The authors suggested that it should be initiated in PCOS women only based on generally accepted criteria for CVD risk [95]. Furthermore, statin’s position in lowering androgen levels compared to other antiandrogenic medications has been compared in a network meta-analysis of randomized clinical trials including 613 patients across 218 articles. They compared the efficacy of statins, metformin, spironolactone, and COC alone or combined in reducing testosterone levels in PCOS. Atorvastatin alone was more effective than COC, spironolactone plus metformin, simvastatin, spironolactone, simvastatin plus metformin, metformin, lifestyle modification, and placebo in reducing the total testosterone level [96]. Besides those positive effects, another interesting point of view is the comparison of ezetimibe and statin. A trial including 28 PCOS patients compared ezetimibe and simvastatin and concluded that although ezetimibe and simvastatin are equipotent in lowering lipid levels in hypercholesterolemic patients with coexisting PCOS, simvastatin exhibits a more pronounced effect on circulating androgen levels in this group [97].

Moreover, other natural medications have been studied in PCOS women; however, the level of evidence for them is even lower. D-chiro-inositol caused a significant reduction in serum testosterone levels and hyperandrogenism, with an improvement in metabolic parameters and ovulation [98]. On the other hand, monacolin K, a naturally derived statin, was suggested to have a similar effect on lipid metabolism as statins, and it was demonstrated to effectively reduce the cholesterol levels in patients with hypercholesterolemia [99]. Inositol alone and the combination of myo-inositol and monacolin K in the treatment of PCOS with insulin resistance, menstrual irregularities, and hirsutism were evaluated in a study by Musacchio et al. Both treatments showed good efficiency, although in the group treated with the combination, improvement in lipids and hyperandrogenism were significantly greater [100]. In another study by Morgante et al., PCOS patients treated with myo-inositol, monacolin K, and lipoic acid were compared with a double dosage of myo-inositol, monacolin K, and lipoic acid for 6 months. Besides the good efficacy of both dosages, the double dosage group showed a significantly greater improvement in terms of lipids and hyperandrogenism [101]. A comparison of these natural medications with statins would be helpful to evaluate their benefits compared to their side effects.

Despite some data on the beneficial effects of statins in the management of women with PCOS, the level of evidence continues to be low due to a lack of studies in this field. Further well-designed RCTs with larger samples in different phenotypes and stages of the disease are needed to support and increase the current level of evidence and help to position the use of statins in PCOS management. In addition, statins are frequently associated with a range of side effects. The most common are muscle-related symptoms such as myalgia, which can affect up to 10% of users [102]. Statins have also been linked to increased liver enzyme levels, indicating potential liver injury, though this is usually asymptomatic and reversible [103]. Furthermore, the weight of evidence suggests that statin use is associated with an increased risk of new-onset diabetes mellitus, but the magnitude of effect has varied across studies and the overall cardiometabolic benefits of statins significantly outweigh this risk. [104]. Additional concern in the PCOS population is that statins are not advised during pregnancy due to the risk of possible teratogenicity [105]. It is therefore essential for clinicians to monitor patients for these adverse effects, balancing the medication’s benefits against its risks.

**Table 2:** Randomized controlled trials exploring the effects of statins on androgen levels.

Study (Refs.)	Population	Statin Used	Dose	Follow-Up	Intervention	Control	Effect on Androgen Levels
[106]	40 patients	Atorvastatin	20 mg	12 weeks	Statin only	Placebo	Decrease *
[107]	84 patients	Simvastatin	20 mg	12 weeks	Statin plus metformin 1500 mg/day	Placebo + metformin 1500 mg/day	Decrease *
[108]	20 patients	Atorvastatin	40 mg	6 weeks	Statin only	Placebo	Decrease *
[109]	97 patients	Simvastatin	20 mg	6 months	Simvastatin plus metformin 1700 mg/day	Simvastatin or metformin 1700 mg/day	Decrease *
[110]	40 patients	Atorvastatin	40 mg	6 weeks	Statin only	Placebo	None
[93]	64 patients	Atorvastatin/Simvastatin	20 mg each	3 months	Atorvastatin	Simvastatin	Decrease in both groups. *
[111]	40 patients	Atorvastatin	20 mg	3 months	Statin only	placebo	Decrease
[112]	64 patients	Simvastatin	20 mg	8 week	Statin only	placebo	Decrease *

* Statistically significant result (*p* less than 0.05).

## 5. Mechanisms of Action of Statins in PCOS

**Figure 2:**
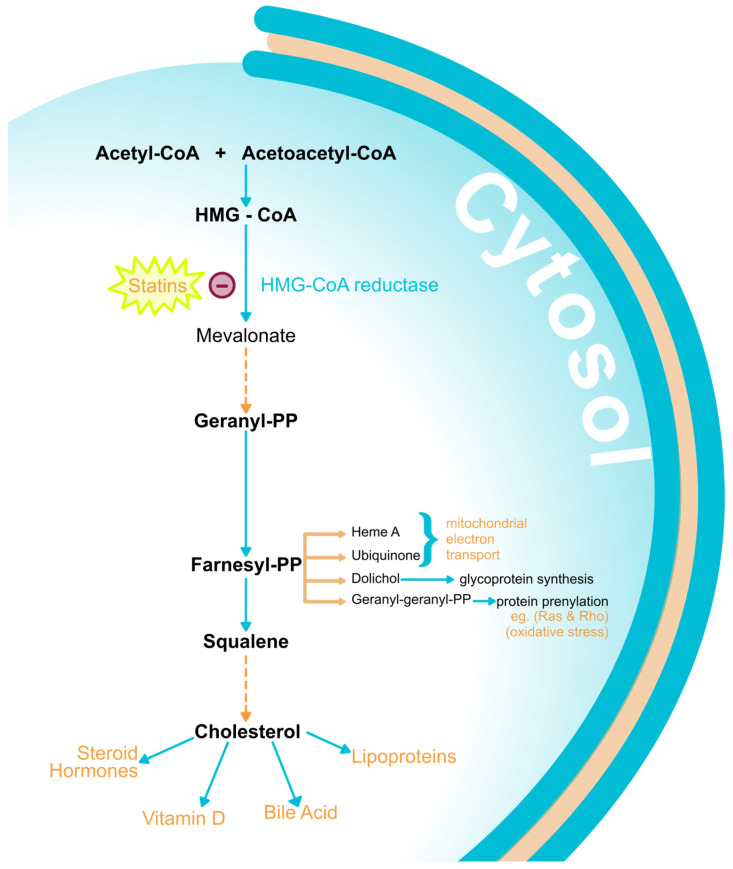
Mevalonate pathway. The upper mevalonate pathway produces mevalonate through the condensation of acetyl-CoA and acetoacetyl-CoA to form 3-hydroxy-3-methyl-glutaryl-Coa (HMG-CoA). The reduction of HMG-CoA through HMG-CoA reductase (substrate for statin inhibition) forms mevalonate. In the lower mevalonate pathway, mevalonate is transformed to geranyl-pyrophosphate through four enzymes and thengeranyl-PP to farnesyl-PP through farnesyl-PP synthase. Farnesyl-PP can then be converted to Heme A, ubiquinone, dolichol, and geranylgeranyl pyrophosphate (GGPP), which carries out the process of protein prenylation with small GTP-binding proteins, i.e., Rho, Ras, and Rac, with inflammatory effects. Finally, farnesyl PP under squalene synthase produces cholesterol in a series of steps, which is a substrate for steroid hormones, vitamin D, and bile acids [117]. Legend: CoA—coenzyme A; HMG-CoA—3-hydroxy-3-methylglutaryl-coenzyme A; HMG-CoA reductase—3-hydroxy-3-methylglutaryl-coenzyme A reductase; PP—pyrophosphate.

Widely known as 3-hydroxy 3-methylglutaryl-CoA (HMG-CoA) reductase inhibitors, statins exhibit lipid-lowering effects through the inhibition of a crucial step in the sterol biosynthetic pathway (Figure 2) [113]. Through the inhibition of the mevalonate pathway, they limit cholesterol biosynthesis, lowering hepatic cholesterol concentrations. Consequently, the expression of LDL receptors in liver cell membranes is raised, facilitating the clearance of LDL with ApoB100 from blood [114]. Statins can also reduce intestinal cholesterol absorption [115] and increase HDL levels [116]. The inhibition of HMG-CoA reductase also decreases the production of other biologically essential products of the mevalonate pathway, including dolichol, geranylgeranyl pyrophosphate (GGPP), and farnesyl pyrophosphate (FPP), and some small GTP-binding proteins, i.e., Rho, Ras, and Rac, with otherwise significant inflammatory and oxidative effects (Figure 2) [117].

**Figure 3:**
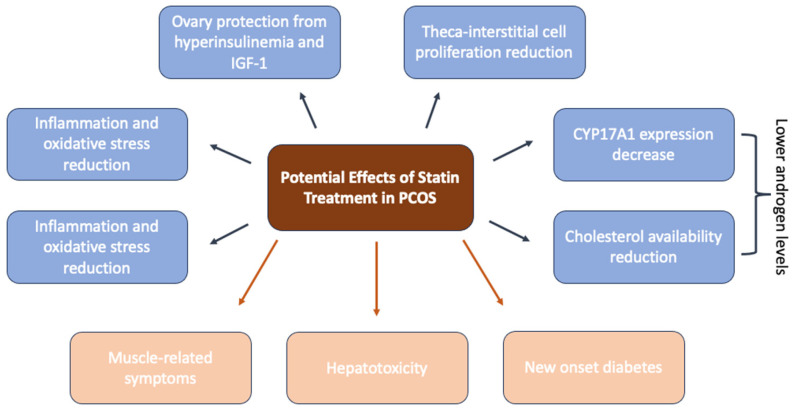
Potential effects of statin treatment in PCOS. Legend: Blue boxes present potential beneficial effects of statins in PCOS and pink boxes present potential harmful effects; CYP17A1—Cytochrome P450 17α-hydroxylase/17,20-lyase; IGF-1—Insulin-like growth factor 1.

In PCOS, where there is a combination of hyperandrogenemia, insulin resistance, and an increased proliferation of theca cells, statins exert positive effects by interfering in different steps of its pathophysiology. To explain how the mevalonate pathway is connected with beneficial outcomes in PCOS, many animal studies have explored the statins’ effects on the inhibition of this pathway and the consequences in different components of PCOS. Figure 3 summarizes these potential beneficial effects [89].

Therefore, by reducing cholesterol synthesis they decrease cholesterol availability, a necessary substrate for steroid hormone synthesis. Moreover, dolichol, a product of the mevalonate pathway (Figure 2), is required for the maturation of insulin and IGF-1 receptors. Therefore, the reduction of dolichol through the inhibition of the pathway from statins can protect the ovaries from excessive levels of insulin and IGF-1, which are critical points in PCOS pathogenesis [89]. Statins are also shown to have beneficial effects on insulin resistance. A meta-analysis involving 406 patients with PCOS evaluated HOMA-IR, fasting glucose, and fasting insulin levels in patients treated with atorvastatin compared to placebo. HOMA-IR showed a significant decrease and fasting insulin was lower in the atorvastatin group, suggesting the amelioration of insulin sensitivity by statins in the PCOS group. However, fasting glucose did not show any difference between the two groups [94]. Even though this conclusion is not one-sided, because statins are also often associated with an increase in diabetes incidence and progression [118], in some PCOS animal models, they are even known to improve insulin resistance [119].

Moreover, statins can reduce the proliferation of theca-interstitial cells through the interruption of the mevalonate pathway [120]. They also have anti-inflammatory properties due to the inhibition of pro-inflammatory cytokines. Simvastatin therapy, for example, significantly reduces IL-2 [121]. In addition, GGPP and FPP (products of the mevalonate pathway shown in Figure 2) play an essential role in the posttranslational modifications of small GTPase proteins (Ras, Rho). These proteins play an important role in the regulation of proliferation, apoptosis, and various other cell functions, including the modulation of reactive oxygen species. In addition, the interruption of prenylation decreases tissue growth and reduces oxidative stress [122]. More evidence is provided in a study where mevastatin showed inhibitory effects on the proliferation of theca-interstitial cells and steroidogenesis [123].

Effects of statins on the enzymatic activity of enzymes involved in steroidogenesis have also been studied in PCOS rats. Inhibitory effect was correlated in a time and concentration manner with a decrease in the mRNA levels of CYP17A1. This finding indicates that the statin-induced reduction in androgen levels is at least partially due to the inhibition of isoprenylation, resulting in a decreased expression of CYP17A1, especially in PCOS, where this enzyme is prominent [124]. Statins have also demonstrated beneficial effects on the suppression of AGE receptors [125], which act as disruptors in PCOS steroidogenesis.

Lastly, proprotein convertase subtilisin/kexin type 9 (PCSK9) concentrations, a protein that increases circulating LDL-C levels by increasing LDL-C receptor degradation, were found to be higher in PCOS patients compared to controls. It is suggested that this abnormally high expression of PCSK9 may be involved in the pathogenesis of PCOS by altering ovarian function and lipid metabolism [126]. However, statins can further raise levels of PCSK9, and this increase can reduce the LDL-C response to statin therapy. This effect can partially explain the limitations of statin therapy to lower LDL-C levels and intra-individual LDL-C response with a nonlinear relationship between statin dose and LDL-C reduction [127]. This limitation of statin therapy opens the possibility of scientific research on PCSK9 modulators and their influence on the physiopathology of PCOS.

## 6. Future Perspectives

PCSK9 modulators are at the forefront of advances in cholesterol metabolism by modulating LDL receptors. These agents facilitate the removal of LDL cholesterol from the circulation, providing a precise strategy for managing hypercholesterolemia and mitigating cardiovascular risk. The PCSK9 inhibitor class includes the monoclonal antibodies alirocumab and evolocumab, as well as the emerging small interfering RNA molecule inclisiran. However, research into the effects of PCSK9 inhibition on the endocrine system remains limited. The majority of available manuscripts describe simple associations between the concentration of PCSK9 and selected endocrine disorders, without exploring causal relationships [128]. However, because of its significant cholesterol-lowering potential, concern over lowering steroidogenesis is present. Data from clinical trials (DESCARTES) showed a lack of impact of evolocumab on estradiol and testosterone levels during a 52-week treatment, suggesting their safety in reproductive function [129]. Moreover, their cardiovascular benefits and the known impact of the cardiovascular system on sexual functions gives additional effects in improving sexual function in patients with familial hypercholesterolemia [130]. Based on low evidence that PCSK9 concentrations are higher in PCOS patients [131] and significantly higher in the liver and ovaries of PCOS patients [126], the possibility of using PCSK9 modulators in PCOS patients is implied. Currently, PCSK9 polymorphisms are studied as a potential risk factor for the development of PCOS [132]. Alirocumab was found to alleviate lipid metabolic disorders, serum reproductive hormones, and other pathological changes in ovarian morphology in PCOS mice [126]. These observations open the possibility of PCSK9 modulator use in PCOS women with a focus on both metabolic profile and hyperandrogenism. However, recent findings from a drug-targeted Mendelian randomization study involving patients with PCOS, premature ovarian insufficiency, premenstrual syndrome, and abnormal uterine bleeding suggest that PCSK9 inhibitors may increase the risk of infertility, a risk profile that appears distinct from that of statins and ezetimibe. Concerns regarding antigenicity and immune responses associated with alirocumab and evolocumab have emerged, underscoring the need for additional research [133].

This review offers a comprehensive examination of the existing literature on the application of statins in the treatment of PCOS, encompassing an analysis of pathophysiology, a synthesis of the available evidence to date, and an exploration of the underlying mechanisms of action and certain limitations. The strength of this review lies in its broad scope, which integrates findings from a variety of studies to present a holistic view of the subject matter. It also attempts to elucidate the complex interactions between statins and the multifaceted endocrine dynamics inherent in PCOS, thereby providing a valuable resource for both clinicians and researchers in the field. Despite these strengths, the review faces inherent limitations. Primarily, the overall quality of evidence currently available is low, characterized by a lack of large-scale, high-quality randomized controlled trials. Furthermore, the scarcity of longitudinal studies addressing long-term outcomes and the paucity of research focusing on diverse populations within the PCOS diagnosis restrict our understanding of the full potential and limitations of statin therapy in this context.

## 7. Conclusions

Current guidelines recommend assessing the lipid profile as an important cardiovascular risk factor in women with PCOS, with suggested screening frequency every 2 years [134]. In addition to their lipid-lowering potential, other beneficial effects in treating hyperandrogenemia make them a promising treatment option for some PCOS patients. However, further research is needed to establish clear recommendations for their use in PCOS management, including which patients would benefit most, when to start treatment, the optimal duration of therapy, and the potential benefits of combining statins with other treatments.

## Figures and Tables

**Table 1:** Apolipoprotein characteristics in PCOS.

Apolipoprotein	Characteristics	Levels in PCOS
ApoA-I	Carried with HDL, cardioprotective effects.	Low [75].
ApoB	Present in all atherogenic lipoproteins.	Elevated in PCOS subgroup related to insulin resistance [76].
ApoC-I	Inhibits uptake of TG-rich lipoproteins via hepatic receptors.	Elevated in PCOS women even with lean patients. Probably the earliest variation in lipid metabolic abnormality in PCOS [77].
ApoE	Present in HDL and LDL.	Low [78].
Lipoprotein a	ApoB-100 and ApoA, atherogenic, prothrombotic.	High * [79].

* Lipoprotein a concentration in women with PCOS is found significantly higher in different studies even in 1/3 of those with a firstly normal lipid profile [58,59].

## Data Availability

Not applicable.
